# Traumatic Brain Injury and Coenzyme Q10: An Overview

**DOI:** 10.3390/ijms26115126

**Published:** 2025-05-27

**Authors:** David Mantle, Mollie Dewsbury, Alexander David Mendelow, Iain P. Hargreaves

**Affiliations:** 1 Pharma Nord (UK) Ltd., Morpeth NE61 2DB, UK; dmantle@pharmanord.co.uk; 2 School of Pharmacy, Liverpool John Moores University, Liverpool L3 5UA, UK; m.r.dewsbury@2024.ljmu.ac.uk; 3 Neurosurgical Trials Group, University of Newcastle upon Tyne, Newcastle upon Tyne NE1 7RU, UK; amendelow@gmail.com

**Keywords:** traumatic brain injury (TBI), mitochondrial dysfunction, oxidative stress, coenzyme Q10, intranasal drug delivery

## Abstract

The incidence of morbidity and mortality in patients who have suffered traumatic brain injury (TBI) is such that novel therapeutic strategies are currently required. There is good evidence that ischaemia is the primary, and sometimes the secondary, cause of brain damage in TBI. This ischaemia may lead to mitochondrial dysfunction, with associated oxidative stress and inflammation, in the pathogenesis of brain injury following head trauma. This, in turn, provides a rationale for the use of supplemental coenzyme Q10 (CoQ10) in the management of TBI, given its key roles in normal mitochondrial function and as an antioxidant and anti-inflammatory agent. In this article, we, therefore, review the use of supplemental CoQ10 in animal models of TBI and its potential application in the management of TBI patients. The problem of blood–brain barrier access is discussed, and how this might be circumvented via the use of an intranasal route to provide direct access of CoQ10 to the brain. In addition, there is evidence that TBI patients have an increased risk of developing cardiac dysfunction and that this may be mediated by aberrant immune action. Given the role of CoQ10 in promoting normal cardiac function and normal immune function, the administration of CoQ10 to prevent cardiovascular complications may improve outcomes in TBI patients.

## 1. Introduction

Traumatic brain injury (TBI) refers to the damage to brain structure and function following exposure of the skull to an external force, for example, following a road traffic accident, and symptoms can vary from concussion to coma and brain death. The severity of a TBI is classified from mild to severe based on the Glasgow Coma Scale. TBIs are broadly divided into skull penetrating and non-penetrating injuries, and into focal or diffuse types, depending on the extent of injury [1]. TBI involves two pathological processes: primary brain injury and secondary brain injury. Primary brain injury refers to brain contusion, subarachnoid haemorrhage, parenchymal haemorrhage, epidural hematoma, and subdural hematoma, which may require surgical intervention; secondary injury involves cerebral oedema and the damaging effects of mitochondrial dysfunction. In both primary and secondary brain damage, ischaemia plays an important role.

Conventional treatment of TBI includes monitoring of hypotension, hypoxia, and intracranial pressure, as well as surgical intervention [2]. However, death and disability rates from TBI remain high, and there are few effective pharmacotherapies (particularly those relating to mitochondrial dysfunction) for TBI patients. Following the initial injury, secondary tissue damage can occur as a result of brain tissue metabolism, particularly following tissue ischaemia/reperfusion. Such metabolic changes include mitochondrial dysfunction, increased oxidative stress associated with increased production of free radical species, and inflammation [3,4]. Coenzyme Q10 (CoQ10) is a vitamin-like substance that has key roles in mitochondrial function and acts as an antioxidant and anti-inflammatory agent. Therefore, the objective of the present article was to review the potential role of CoQ10 in the management of traumatic head injury.

## 2. Mitochondrial Dysfunction in Traumatic Brain Injury

The brain uses more energy than any other organ, accounting for approximately 20% of the total body energy requirement. To provide the energy required, brain tissue contains high levels of mitochondria, which are found in all brain cell types. A major cause of TBI-associated brain damage is secondary injury, which, in turn, results principally from mitochondrial dysfunction [5,6]. Mitochondrial injury leads to decreased energy supply, altered mitochondrial permeability transition pore function, calcium dysregulation, oxidative stress, and inflammation [7]. Evidence from studies in animal models of TBI suggests that these metabolic changes occur relatively early (within hours) after the primary injury [8]. Such cellular alterations, together with the release of apoptogenic proteins from mitochondria into the cytosol, serve as a primary mechanism responsible for inducing apoptosis, leading to the impaired neurologic functioning that has been observed in individuals with TBI.

Morphologically, the determination of mitochondrial dysfunction after human TBI is limited by the availability of tissue removed from living patients and rapidly processed to minimise *post-mortem* and ex vivo alterations. Balan et al. [9] quantified mitochondrial morphologic alterations in surgically resected and rapidly fixed human TBI tissue, characterising mitochondrial ultrastructural changes at progressive distances from the injury site.

The morphological changes in TBI are proportional to mitochondrial dysfunction and microglial activation [10]. Microglia and brain-infiltrating macrophages are responsible for the production of inflammatory cytokines. In addition to their role in providing energy support, mitochondria reprogram the pro- and anti-inflammatory machinery in immune cells. Microglial pro-inflammatory activation is associated with decreased mitochondrial respiration, whereas anti-inflammatory microglial polarisation is supported by increased oxidative metabolism; thus, mitochondria are central regulators of post-traumatic neuroinflammation [11].

Both experimental and clinical TBI studies have documented mitochondrial dysfunction. Following TBI, a decline in ATP production is demonstrable in both rats and humans at a time when energy requirements increase; decreased ATP production and increased lactate accumulation are hallmarks of mitochondrial dysfunction. Evidence for mitochondrial dysfunction following TBI can be obtained from a number of sources; in patients, these include cerebral microdialysis sampling, positron emission tomography (PET), and magnetic resonance spectroscopy [12,13,14,15,16].

For example, in a study by Verweij et al. [17], mitochondria derived from therapeutically removed brain tissue were analysed in 16 patients with head injury (Glasgow Coma Scale Scores of 3–14) and two patients without head injury. Their results revealed that in patients with head injury, mitochondrial function was impaired, with subsequent decreased ATP production. Wu et al. [18] reported the mitochondrial deubiquitinase USP30 to be upregulated after TBI in humans and mice; knockout of USP30 reduced lesion volumes, mitigated brain oedema, and attenuated neurological deficits after TBI in mice. Additionally, USP30 deficiency effectively suppressed oxidative stress and neuronal apoptosis in TBI. The protective effects of USP30 loss were thought to be attributed, at least partially, to the reduction in TBI-induced impairment of mitochondrial quality control, including mitochondrial dynamics, function, and mitophagy.

By monitoring the blood levels of mitochondrial proteins, Sinha et al. [19] found Complex I, Complex IV and pyruvate dehydrogenase to be predictors of long-term outcomes in severe traumatic brain injury. Further evidence of mitochondrial dysfunction was demonstrated by an elevated lactate/pyruvate ratio following cerebral microdialysis of patients following TBI [20]. Following paediatric TBI, the levels of mitochondrial heat shock protein hsp60 increased in the CSF. The induction of hsp60 occurred early after the injury, and its levels were associated with the severity of injury, defined as the admission Glasgow Coma Scale Score [21]. There is evidence that mitochondrial DNA polymorphisms may impact patient outcomes after TBI, potentially by influencing mitochondrial function [22].

There are four main preclinical animal models of TBI: the fluid percussion injury model, the weight drop injury model, the controlled cortical impact injury, and the blast/diffuse brain injury model [23]. Most of the animal studies referenced in the present article refer to the weight drop or cortical impact models. A number of animal studies have demonstrated changes in mitochondrial morphology following TBI. For example, Wu et al. [24], using the weight drop model of TBI in mice, reported abnormal mitochondria in neurons 24 h after TBI. These abnormalities included severe mitochondrial fragmentation, crista collapse, mitochondrial swelling, mitochondrial membrane rupture, decreased mitochondrial density, and increased size and shape heterogeneity. Similarly, Wiley et al. [25], using a cortical impact mouse model, found the number and volume of mitochondria in the neurons in the damaged area to be decreased.

At the biochemical level, the occurrence of aberrant mitochondrial metabolism in a rat model of acute TBI was monitored by detecting [^13^ C] bicarbonate production from hyperpolarised [1-^13^ C] pyruvate, suggesting that [^13^ C] bicarbonate is a sensitive in vivo biomarker of the secondary injury processes [26]. In a rat model of TBI, Xiong et al. [27] demonstrated excessive Ca^2+^ adsorption to the mitochondrial membrane, which subsequently inhibited the respiratory chain-linked electron transfer and energy transduction. Gilmer et al. [28] reported evidence for mitochondrial bioenergetic deficit relatively early (one hour after injury) in a rat model of head injury.

Mitochondria in the brain are classified as synaptic or non-synaptic; mitochondria located in the neural synapse may undergo the highest bioenergetic demand in the brain as they supply the energy needed during neurotransmission [29]. Synaptic mitochondria are highly involved in the regulation of neurotransmitter release and synaptic vesicle formation [30]. Hill et al. [31] found synaptic mitochondria to be more vulnerable to TBI than non-synaptic mitochondria in terms of damage to mitochondrial proteins and respiratory deficit. Opii et al. [32] identified oxidative damage/reduced activity of a number of mitochondrial proteins in a rat model of TBI, including pyruvate dehydrogenase, ATP synthase, Complex I, and Complex IV. Similarly, Chen et al. [33] found reduced levels of Complexes I, III, and V following TBI in rats. There is evidence that Complex IV may be particularly susceptible to TBI-induced damage [34]. Following TBI in mice, subsequent cerebral cortex oxidative stress and neuronal apoptosis could be ameliorated via over-expression of Uqcr11, a subunit of Complex III [35].

A number of studies have focused on mitochondria as therapeutic targets in TBI. Thus, therapies that reverse mitochondrial uncoupling, increase mitochondrial antioxidant production, or inhibit mitochondrial permeability transition pores (MPTPs) have been investigated [36]. Examples in rodent models of TBI include the restoration of mitochondrial dysfunction (in terms of electron transfer, energy coupling capacity, and Ca^2+^ transport capacity) following the post-injury administration of the antioxidant U-101033E [27]; the attenuation of mitochondrial dysfunction following the inhibition of mitochondrial permeability transition by cyclosporin A [37]; and the restoration of mitochondrial bioenergetics and oxidative balance following the administration of the mitochondrial uncoupling agent 2,4 dinitrophenol [38]. The role of CoQ10 in mitochondrial energy generation was reviewed by Mantle et al. [39].

## 3. Oxidative Stress in Traumatic Brain Injury

Although certain species of free radicals at lower levels can act as signalling molecules, in general terms, free radicals, with their potential to damage cellular components, are continually generated as unwanted by-products of normal cell metabolism. The vulnerability of the brain to oxidative stress (the imbalance between free radical generating and protective systems) results from a number of factors, particularly its high lipid content and relatively modest levels of protective antioxidants [40]. Although free radicals can be generated in various subcellular locations, mitochondria are the major intracellular source of free radical generation, particularly via the leakage of electrons from the electron transport chain during the process of oxidative phosphorylation. In addition to being a major source of free radical generation, mitochondria are also a major target for free radical-induced damage. For example, in contrast to nuclear DNA, mitochondrial DNA has no nucleotide-excision repair pathways and is not protected by histones; mitochondrial DNA is, therefore, particularly prone to mutations, resulting in a bioenergetic deficit where ATP production markedly decreases and free radical production significantly increases. As noted in the previous section, TBI is associated with mitochondrial dysfunction, which, in turn, results in exacerbated free radical generation, resulting in a self-reinforcing cycle of mitochondrial damage and free radical production.

Evidence of oxidative stress associated with TBI has been obtained from both preclinical and clinical studies using various analytical techniques. For example, in a rat model of TBI, Awasthi et al. [41] demonstrated increased free radical production via electron spin resonance spectroscopy. Similarly, Tyurin et al. [42] used electron spin resonance spectroscopy, together with analysis of the lipid peroxidation biomarker 8-epi-prostaglandin F(2alpha), to demonstrate increased oxidative stress in a rat model of TBI. In TBI patients, Yen et al. [43] reported increased levels of the lipid peroxidation biomarkers F(2)-isoprostanes (F(2)-IsoPs) and F(4)-neuroprostanes (F(4)-Nps) in the CSF and plasma, demonstrating enhanced oxidative damage in the brain of TBI patients and the association between higher CSF levels of F(2)-IsoPs and a poor outcome. The antioxidant action of CoQ10 was reviewed by Gutierrez-Mariscal et al. [44]. The role of the antioxidant CoQ10 in the management of TBI is addressed in the following sections of this article.

## 4. Inflammation in Traumatic Brain Injury

There is a common misconception that inflammation, which involves the release of pro-inflammatory cytokines, is a wholly negative process within the body. However, inflammation is the body’s normal response to infection or injury, and it is essential for tissue healing, although this process should resolve following the initial immune response. Thus, after TBI, inflammation could potentially have beneficial effects, provided the process is not too intense or prolonged. Within minutes of impact, the release of damage-associated molecular patterns (DAMPs) prompts resident cells to secrete cytokines, which, in turn, attract neutrophils, thus promoting the removal of damaged and dead cells and debris. Infiltrating monocytes and activated glia begin to predominate 3–5 days post-injury to defend against infection and perform reparative functions. At 3–7 days post injury, T and B cells can also be recruited to the site of injury [45]. When control of this process is lost, further tissue damage contributes to the subsequent neurological deficit and neurodegenerative changes associated with TBI; mitochondrial dysfunction and oxidative stress have been identified as factors contributing to the loss of control of the latter process [46]. CoQ10 performs a number of cellular functions of potential relevance to the immune system; in particular, CoQ10 is able to directly modulate the action of genes involved in inflammation and may have a role in controlling the release of pro-inflammatory cytokines [47].

## 5. Apoptosis, Ferroptosis, and Traumatic Brain Injury

Apoptosis is a process of programmed cell death, which can occur after a number of triggering events, one of which is TBI; inhibition of apoptosis could potentially reverse its deleterious effects and lead to better functional outcomes [48]. Various strategies to inhibit apoptosis have been described, for example, the inhibition of caspase enzymes (as the principal mediators of cell death due to apoptosis) [49]; however, to date, there has been little work to investigate the potential role of CoQ10 in inhibiting apoptosis after TBI. In this regard, the study by Lin et al. [35] is of note, where the upregulation of ubiquinol–cytochrome c reductase, complex III subunit XI (Uqcr11), in a mouse model of TBI reduced neuronal apoptosis. A considerable number of preclinical studies have been reported in which the administration of CoQ10 inhibited apoptosis in disorders other than TBI, for example, after spinal cord injury in rats [50] and in a mouse cell model of diabetes [51].

Ferroptosis is a type of iron-dependent programmed cell death (distinct from apoptosis) of relevance to the pathology of TBI [52]. In animal models of TBI, beneficial effects have been reported following the administration of a number of inhibitors of ferroptosis, although, to date, these do not include CoQ10 [52]. Several preclinical studies on disorders other than TBI have demonstrated the action of CoQ10 or its structural analogues in inhibiting ferroptosis; these include models of epilepsy [53], subarachnoid haemorrhage [54], myocardial infarction [55], Parkinson’s disease [56], and acute liver injury [57].

## 6. CoQ10 Supplementation in Animal Models of Traumatic Brain Injury

Whilst CoQ10 is the predominant (>99%) CoQ form in humans, rat tissues predominantly contain the CoQ9 form; the structure of CoQ9 is very similar to CoQ10, with nine repeating isoprene units in the long isopenoid tail attached to the quinone ring, as opposed to ten isoprene units in the corresponding CoQ10 molecule. The exception is rat brain tissue, which contains approximately 30% of total CoQ in the form of CoQ10. On this basis, the rat model of brain injury is considered to be a valid system for extrapolation of data to humans [58].

Lazzarino et al. [58] found that severe TBI reduced CoQ10 levels in the rat brain compared to rats with moderate TBI or controls. In rats with induced traumatic brain injury, Kalayci et al. [59] showed that the administration of CoQ10 after trauma reduced subsequent tissue damage, quantified in terms of vascular congestion, neuronal loss, nuclear pyknosis, nuclear hyperchromasia, cytoplasmic eosinophilia, and axonal oedema. In a rat model of TBI, the intra-arterial administration of ubiquinol (the reduced form of CoQ10) either 30 min before or after primary injury reduced brain mitochondrial damage, apoptosis, and two serum biomarkers of TBI severity: glial fibrillary acidic protein and ubiquitin C-terminal hydrolase-L1 [60]. The same research group [61] had previously shown that the administration of ubiquinol prior to TBI in rats modified the cerebral expression of genes involved in bioenergetics and oxidative/nitrosative stress. In a rat model of brain injury induced following exposure to potassium dichromate, the oral administration of CoQ10 (50 mg/kg for 3 days) reduced biomarkers of oxidative stress and inflammation and ameliorated cognitive impairment [62]. In a rat model of brain injury induced via exposure to lipopolysaccharide, the oral administration of CoQ10 (200 mg/kg for 7 days prior to injury) decreased markers of brain tissue oxidative stress [63].

In a mouse model of TBI, the administration of the CoQ10 analogue mitoquinone (MitoQ) 30 min post-injury enhanced neurological and cognitive functions 30 days post-injury. MitoQ also decreased the activation of astrocytes and microglia, which was accompanied by improved axonal integrity and neuronal cell count in the cortex. The study demonstrated that MitoQ has neuroprotective effects in a TBI mouse model by decreasing oxidative stress, neuroinflammation, and axonal injury [64]. Similarly, Tabet et al. [65] showed that the administration of MitoQ in a mouse model of TBI reduced astrocytosis, microgliosis, and dendritic and axonal shearing, increased the expression of antioxidant enzymes, and improved motor function and learning impairments. The administration of thymoquinone, a plant-derived compound chemically similar to CoQ10, following TBI in rats reduced oxidative stress levels and improved neuronal survival [66].

## 7. Transport of Coenzyme Q10 Across the Blood–Brain Barrier in Humans

At present, the ability of CoQ10 to cross the human blood–brain barrier (BBB) has yet to be established. In view of this, synthetic analogues of CoQ10, such as idebenone or mitoquinone, have been developed with the intention of improving BBB transport; however, the efficacy and safety of such compounds have yet to be fully established in clinical studies [67]. To elucidate the mechanisms by which CoQ10 may cross the BBB, Wainwright et al. [68] utilised an in vitro porcine endothelial model of the barrier and identified lipoprotein-associated CoQ10 transcytosis in both directions across the BBB. CoQ10 uptake via SR-B1 (Scavenger Receptor) and RAGE (Receptor for Advanced Glycation End products) receptors was matched by efflux via LDLR (Low-Density Lipoprotein Receptor) transporters, resulting in no “net” transport across the BBB. When CoQ10 deficiency was induced in the model (using p-aminobenzoic acid, a competitive inhibitor of COQ2), BBB tight junctions were disrupted, and CoQ10 “net” transport to the brain side increased. The in vitro porcine BBB model was also used to investigate the transport of idebenone across the BBB, which was found to have a much greater transport from the blood to the brain side of the barrier than CoQ10, being able to cross the barrier directly rather than requiring incorporation into a lipoprotein complex [68]. It has been suggested that the uptake of exogenous CoQ10 into the brain may be improved by the administration of LDLR inhibitors or by the stimulation of luminal SR-B1 transporters; however, this has yet to be undertaken experimentally [68].

## 8. Intranasal Delivery of CoQ10 in Traumatic Brain Injury

A major obstacle to the successful delivery of therapeutic drugs to the central nervous system is the BBB [69]. As noted in the previous section of this article, preclinical studies have indicated that CoQ10 may be able to access the BBB, but this has not been established in clinical studies [70]. The disappointing outcome of clinical trials supplementing CoQ10 in a number of neurological disorders may be related to the inability of CoQ10 to cross the BBB in humans; however, corresponding studies in animal models have shown therapeutic promise [71,72]. Although a restricted class (molecular weight < 400 Da) of lipid-soluble drugs can freely access the BBB, the latter prevents 98% of small and 100% of large molecules from entering the brain [73]. Although intracerebroventricular and intraparenchymal routes may provide the effective delivery of such substances in preclinical models, clinically, these delivery methods are invasive and risk inadequate exposure to injured brain regions because of the rapid turnover of cerebrospinal fluid.

**Figure 1:**
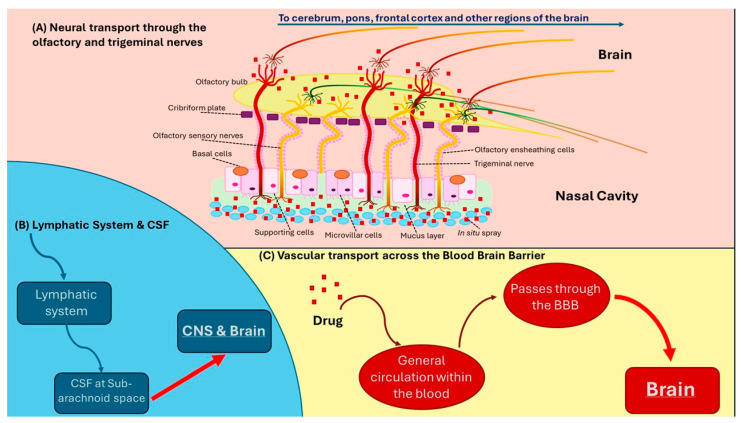
Neural transport through the olfactory and trigeminal nerve to the brain (**A**) and through the lymphatic system and subarachnoid space to the brain (**B**) + vascular transport across the blood–brain barrier to the brain (**C**). CSF: cerebrospinal fluid.

The delivery of drugs directly to the brain via an intranasal route, initially developed by Frey [74], represents a non-invasive method for bypassing the BBB, essentially free of adverse effects. Intranasal drug delivery to the brain is facilitated by the olfactory and trigeminal nerves (Figure 1). The intranasal route comprises two pathways: one intracellular and one extracellular. The intracellular pathway involves endocytosis by olfactory sensory cells, followed by axonal transport to their synaptic clefts in the olfactory bulb, where the drug is exocytosed. This trans-synaptic process is repeated by olfactory neurons, facilitating drug delivery to other brain regions. In the extracellular pathway, drugs are transported directly into the CSF by first transiting the paracellular space across the nasal epithelium, then via the perineural space into the subarachnoid space of the brain [75].

Candidate drugs for intranasal delivery should be readily dissolved in the vehicle solvent, permeable to the nasal mucosa, and meet clinical criteria for safe delivery. Drugs with lower molecular weight and higher lipophilicity generally favour rapid intranasal uptake and brain delivery; in this regard, it is of note that CoQ10 is one of the most lipophilic naturally occurring substances. Additional critical pharmacological factors that dictate the bioavailability and efficacy of intranasal compounds include drug metabolism in the nasal cavity, degree of dissociation (pKa), chemical structure, drug half-life, osmolarity, and pH. The delivery of compounds to the nasal mucosa may be hindered by the action of proteolytic enzymes located within the latter [76]. Table 1 outlines previous randomised clinical trials that have utilised intranasal delivery to bypass the BBB.

In animal models of traumatic brain injury, a number of compounds of relevance to mitochondrial function, either for their role in energy metabolism, as antioxidants, or with other functions, can be delivered directly to the brain via the intranasal route (reviewed by Pandya et al. [76]). 

Of particular interest is the study by Silachev et al. [90], in which brain injury in rats was induced via middle cerebral artery occlusion. The intranasal administration (1 umol/kg) of the CoQ10 analogue SKQ1 [67] showed a high level of penetration into brain tissue, with a concomitant reduction in tissue injury. The use of intranasal DDAVP (1-deamino-8-D-arginine vasopressin) was described successfully by Kauli and Laron in 1974 [91], and this pathway has been routinely used since [92,93]. Table 2 summarises therapeutic targets and preclinical study outcomes for CoQ10 supplementation in TBI.

No clinical studies were identified in which supplemental CoQ10 was administered specifically to TBI patients. However, Hasanloei et al. [94] described a randomised controlled trial involving 40 trauma patients (type of trauma not specified) with low plasma CoQ10 levels admitted to the ICU. The sublingual administration of CoQ10 (400 mg/day for 7 days) resulted in significant reductions in oxidative stress and inflammatory biomarkers (malondialdehyde, interleukin-6), Glasgow Coma Score, ICU and hospital length of stay, and mechanical ventilation duration. In addition, CoQ10 administration increased fat-free mass and skeletal muscle mass. The use of supplemental CoQ10 in combination with selenium in ICU patients was reviewed by Hargreaves and Mantle [95].

To date, no studies, either preclinical or clinical, have been carried out to investigate the potential delivery of CoQ10 directly to the brain via the intranasal route, and this is an area identified by the authors as requiring further research.

## 9. Conclusions

1. As noted in the Introduction, the incidence of morbidity and mortality in patients who have suffered TBI is such that novel therapeutic strategies would be desirable. We reviewed evidence for the involvement of mitochondrial dysfunction, with associated oxidative stress, inflammation, and apoptosis/ferroptoses, in the pathogenesis of brain injury following head trauma.

2. Based on the studies identified in item 1 above, there is a rationale for the use of supplemental CoQ10 in the management of TBI, given its key roles in normal mitochondrial function, as an antioxidant, anti-apoptotic/ferroptotic, and anti-inflammatory agent (summarised in Table 2).

3. A number of studies using animal models of TBI have described beneficial outcomes following supplementation of CoQ10 or its structural analogues (summarised in Table 2).

4. Based on the studies identified in item 3 above, there is a rationale for the supplementation of CoQ10 in patients with TBI. However, to date, no clinical studies have been carried out to assess the potential benefit of CoQ10 supplementation in TBI patients. Two potential problems with this approach are the low bioavailability of orally administered CoQ10 [96] and whether supplemental CoQ10 can cross the blood–brain barrier in humans. Some attempts to overcome the poor bioavailability of orally administered CoQ10, at least in animal models, have been described using an intravenous injection route [97]; however, this approach does not address the problem of BBB accessibility. There is evidence for the disruption of the BBB in some cases of TBI [98], and it is difficult to predict the effect of such disruption with regard to the transit of CoQ10. 

5. A possible method to circumvent the difficulty of BBB access is the administration of CoQ10 directly into the brain via the intranasal route. The efficacy and safety of this route to administer prescription-type drugs of relevance to neurological function directly into the brain have been established (summarised in Table 1). However, to date, no clinical studies have investigated the potential of this route of administration for CoQ10. This is an area identified by the authors as requiring future research. In this regard, a recent study by Capossela et al. [99] is of note, in which nerve growth factor administered via the intranasal route to an adolescent with severe TBI resulted in a significant improvement in clinical outcomes.

6. In addition to the above area of research, there is evidence that TBI patients have an increased risk of developing cardiac dysfunction [100] and that this may be mediated by aberrant immune action [101]. Given the role of CoQ10 in promoting normal cardiac function [102] and normal immune function [47], the administration of CoQ10 to prevent cardiovascular complications in TBI patients remains another area for future research. In general terms, the safety of CoQ10 supplementation is well established. More than 200 randomised controlled clinical trials have been listed to date on Medline, in which CoQ10 has been supplemented in a wide range of disorders. Dosage regimes on the order of 200–300 mg/day for 3–6 months are typically utilised, although some studies have used much higher daily doses (2700 mg/day) or longer intervention periods (up to 5 years). None of these studies has reported any serious adverse effects as a result of CoQ10 supplementation. Finally, although this article focused on the potential benefits of CoQ10 supplementation in TBI, the potential benefits of supplementation with other mitochondrial metabolites should be considered. Beneficial results have been reported in animal models of TBI following supplementation with, for example, NAD [103], alpha-lipoic acid [104], and acetyl-L-carnitine [105]; however, no clinical studies supplementing these metabolites in TBI have been reported, and these constitute other areas for future research.

## Figures and Tables

**Table 1:** Randomised clinical trials using intranasal drug delivery.

Drug	Indication	Outcome	Reference
Sumatriptan	Migraine	Greater relief in pain intensity versus oral delivery	Tepper et al. (2015) [77]
Sumatriptan	Migraine	Faster reduction in pain intensity versus oral delivery	Lipton et al. (2017) [78]
Sumatriptan	Migraine	Reduced nausea versus oral delivery	Lipton et al. (2018) [79]
Insulin	Alzheimer’s disease	Improved cognition	Claxton et al. (2015) [80]
Insulin	Alzheimer’s disease	Reduced white matter hyperintensity volume progression	Kellar et al. (2021) [81]
Insulin	Alzheimer’s disease	Reduced levels of CSF inflammatory markers	Kellar et al. (2022) [82]
Midazolam	Sedative	Improved bioavailability versus intravenous delivery	Bancke et al. (2015) [83]
Midazolam	Neonatal sedation	More effective than ketamine	Milesi et al. (2018) [84]
Diazepam	Anticonvulsant	More acceptable administration route versus rectal delivery	Henney et al. (2014) [85]
Lorazepam	Anticonvulsant	Less invasive alternative to intramuscular injection	Ahmad et al. (2006) [86]
Esketamine	Treatment resistant depression	Efficacy and safety confirmed	McIntyre et al. (2024) [87]
Tizanidine	Muscle spacticity	Greater bioavailability versus oral delivery	Vitale et al. (2013) [88]
Scopolamine	Motion sickness	More rapid absorption versus oral or transdermal delivery	Simmons et al. (2010) [89]

**Table 2:** Summary of therapeutic targets and preclinical study outcomes for CoQ10 supplementation in TBI.

Study	Outcome	Case Model
Yonutas et al., 2016 [36]	Reviewed the therapeutic approaches to ameliorate mitochondrial dysfunction following brain injury.	Review
Sullivan et al., 2005 [37]	Reviewed the evidence relating to mitochondrial permeability transition in central nervous system trauma and evidence for therapeutically targeting the mitochondrial permeability transition in TBI.	Review
Hubbard et al., 2023 [38]	Reported that mild mitochondrial uncoupling can restore mitochondrial bioenergetics and oxidative balance following TBI.	Animal model using male Sprague Dawley rats at 8 weeks of age; there were six experimental groups, each with eight subjects. These groups were subjected to compressed helium-driven blasts at 11 psi to induce mTBI. The treatment groups received 8 or 80 mg/kg of MP201 (2,4-dinitrophenol prodrug, uncoupler) with administration early or delayed after mTBI.
Yen et al., 2015 [43]	Reported increased levels of lipid peroxidation biomarkers and the need for antioxidant protection in TBI patients.	Moderate and severe TBI patients with an age range of 15–75 years. The patients were randomly treated with 10 mg/mL propofol or 5 mg/mL midazolam for 72 h postoperation. Cerebrospinal fluid and plasma were collected from 15 patients for 6–10 days after exposure.
Simon et al., 2017 [46]	Found that mitochondrial dysfunction and oxidative stress contributed to the loss of control of the inflammation process in TBI patients. The loss of control of this process resulted in further tissue damage, neurological deficit, and neurodegenerative changes associated with TBI.	Review
Mantle et al., 2021 [47]	Reported that CoQ10 could modulate directly the action of genes involved in inflammation and may help to control the release of pro-inflammatory cytokines.	Review
Lin et al., 2023 [35]	Reported that the upregulation of ubiquinol–cytochrome c reductase, complex III subunit XI (Uqcr11), in a mouse model of TBI reduced neuronal apoptosis.	Review
Geng et al., 2021 [52]	Reported that ferroptosis was related to the pathology of TBI and that the inhibition of ferroptosis could improve long-term outcomes of TBI.	Review
Fikry et al., 2023 [53]	A rat study found that CoQ10 had a beneficial targeted effect on hippocampal oxidative stress and ferroptosis.	A lithium–pilocarpine rat model was created using male Wistar rats from 6 to 8 weeks old. Seizures were induced using 0.5 mg/mL pilocarpine diluted in DMSO, injected intraperitoneally at 100 mg/kg. The CoQ10-treated group was given CoQ10 at 20 mg/kg via gavage once a day for 2 weeks before it was given a Pilo injection.
Lazzarino et al., 2023 [58]	A rat study found that severe TBI changed the levels and redox states of CoQ9 and CoQ10, indicating TBI-associated mitochondrial impairment affecting oxidative phosphorylation, energy generation, and antioxidant defence.	Rat study with induced graded TBIs (mild, moderate, and severe) in 26 male Wistar rats of 300–350 g body weight (b.w.). The subjects were administered 35 mg/kg b.w. ketamine and 0.25 mg/kg b.w. midazolam to indue anaesthesia prior to TBI induction. Reduced and oxidised CoQ9 and CoQ10 were determined via HPLC analysis.
Kalayci et al., 2011 [59]	A rat study found that CoQ10 decreased neuronal degeneration, secondary brain damage, and ischemia caused by oxidative stress in TBI rats.	The study used 28 Wistar albino male rats with a body weight between 350 and 400 g to create a brain injury model. The rats in the CoQ10 group were administered a CoQ10 dose of 10 mg/kg immediately after trauma was induced and again at the 24th hour post-trauma via gavage.
Pierce et al., 2018 [60]	A rat study found that ubiquinol administered before or after TBI reduced brain mitochondrial damage, apoptosis, and serum biomarkers of TBI severity.	The study used 36 adult male F344 rats with induced TBI. The rats with pre-treatment before TBI were administered 100 mg/kg b.w. ubiquinol intra-arterially 30 min before the cortical impact. The rats with post-TBI treatment were administered 100 mg/kg b.w. ubiquinol 30 min after the cortical impact.
Salama et al., 2021 [62]	A rat study found that CoQ10 reduced biomarkers of oxidative stress and inflammation in a rat model of brain injury.	Rat model with potassium dichromate (PD) induced brain injury via the intranasal administration of 2 mg/kg PD. Male Wister albino rats of 140–150 g were used. Starting 24 h post-PD-induced brain injury, the subjects were administered 50 mg/kg CoQ10 orally for 3 days. Prior to and after the experiment, locomotor activity was assessed, and biochemical and histopathological investigations were assessed in the brain homogenate.
El-Laithy et al., 2018 [63]	A rat study found that CoQ10 decreased biomarkers of brain tissue oxidative stress in a rat model of brain injury.	The study used 66 female Wistar albino rats with a body weight between 100 and 120 g at 3 months old. The groups were treated with 100 mg/kg or 200 mg/kg CoQ10. The subjects were treated with or without lipopolysaccharide (LPS) simultaneously with CoQ10 to induce brain injury.

## References

[1] Orr T.J., Lesha E., Kramer A.H., Cecia A., Dugan J.E., Schwartz B., Einhaus S.L. (2024). Traumatic Brain Injury: A comprehensive review of biomechanics and molecular pathophysiology. World Neurosurg..

[2] Galgano M., Toshkezi G., Qiu X., Russell T., Chin L., Zhao L.R. (2017). Traumatic brain injury: Current treatment strategies and future endeavors. Cell Transplant..

[3] Hiebert J.B., Shen Q., Thimmesch A.R., Pierce J.D. (2015). Traumatic brain injury and mitochondrial dysfunction. Am. J. Med. Sci..

[4] Hakiminia B., Alikiaii B., Khorvash F., Mousavi S. (2022). Oxidative stress and mitochondrial dysfunction following traumatic brain injury: From mechanistic view to targeted therapeutic opportunities. Fundam. Clin. Pharmacol..

[5] Cheng G., Kong R.H., Zhang L.M., Zhang J.N. (2012). Mitochondria in traumatic brain injury and mitochondrial-targeted multipotential therapeutic strategies. Br. J. Pharmacol..

[6] Demers-Marcil S., Coles J.P. (2022). Cerebral metabolic derangements following traumatic brain injury. Curr. Opin. Anaesthesiol..

[7] Benaroya H. (2020). Brain energetics, mitochondria, and traumatic brain injury. Rev. Neurosci..

[8] Jiang X.B., Ohno K., Qian L., Tominaga B., Kuroiwa T., Nariai T., Hirakawa K. (2000). Changes in local cerebral blood flow, glucose utilization, and mitochondrial function following traumatic brain injury in rats. Neurol. Med. Chir..

[9] Balan I.S., Saladino A.J., Aarabi B., Castellani R.J., Wade C., Stein D.M., Eisenberg H.M., Chen H.H., Fiskum G. (2013). Cellular alterations in human traumatic brain injury: Changes in mitochondrial morphology reflect regional levels of injury severity. J. Neurotrauma.

[10] Kumar Sahel D., Kaira M., Raj K., Sharma S., Singh S. (2019). Mitochondrial dysfunctioning and neuroinflammation: Recent highlights on the possible mechanisms involved in Traumatic Brain Injury. Neurosci. Lett..

[11] Strogulski N.R., Portela L.V., Polster B.M., Loane D.J. (2023). Fundamental neurochemistry review: Microglial immunometabolism in traumatic brain injury. J. Neurochem..

[12] Signoretti S., Marmarou A., Aygok G.A., Fatouros P.P., Portella G., Bullock R.M. (2008). Assessment of mitochondrial impairment in traumatic brain injury using high-resolution proton magnetic resonance spectroscopy. J Neurosurg..

[13] Nordström C.H., Nielsen T.H., Schalén W., Reinstrup P., Ungerstedt U. (2016). Biochemical indications of cerebral ischaemia and mitochondrial dysfunction in severe brain trauma analysed with regard to type of lesion. Acta Neurochir..

[14] Thelin E.P., Carpenter K.L., Hutchinson P.J., Helmy A. (2017). Microdialysis monitoring in clinical traumatic brain injury and Its role in neuroprotective drug development. AAPS J..

[15] Khellaf A., Garcia N.M., Tajsic T., Alam A., Stovell M.G., Killen M.J., Howe D.J., Guilfoyle M.R., Jalloh I., Timofeev I. (2022). Focally administered succinate improves cerebral metabolism in traumatic brain injury patients with mitochondrial dysfunction. J. Cereb. Blood Flow. Metab..

[16] Svedung Wettervik T., Hånell A., Howells T., Enblad P., Lewén A. (2022). Females exhibit better cerebral pressure autoregulation, less mitochondrial dysfunction, and reduced excitotoxicity after severe traumatic brain injury. J. Neurotrauma.

[17] Verweij B.H., Muizelaar J.P., Vinas F.C., Peterson P.L., Xiong Y., Lee C.P. (2000). Impaired cerebral mitochondrial function after traumatic brain injury in humans. J. Neurosurg..

[18] Wu Y., Hu Q., Cheng H., Yu J., Gao L., Gao G. (2023). USP30 impairs mitochondrial quality control and aggravates oxidative damage after traumatic brain injury. Biochem. Biophys. Res. Commun..

[19] Sinha S., Raheja A., Samson N., Bhoi S., Selvi A., Sharma P., Sharma B.S. (2016). Blood mitochondrial enzymatic assay as a predictor of long-term outcome in severe traumatic brain injury. J. Clin. Neurosci..

[20] Vespa P., Bergsneider M., Hattori N., Wu H.M., Huang S.C., Martin N.A., Glenn T.C., McArthur D.L., Hovda D.A. (2005). Metabolic crisis without brain ischemia is common after traumatic brain injury: A combined microdialysis and positron emission tomography study. J. Cereb. Blood Flow Metab..

[21] Lai Y., Stange C., Wisniewski S.R., Adelson P.D., Janesko-Feldman K.L., Brown D.S., Kochanek P.M., Clark R.S. (2006). Mitochondrial heat shock protein 60 is increased in cerebrospinal fluid following pediatric traumatic brain injury. Dev. Neurosci..

[22] Conley Y.P., Okonkwo D.O., Deslouches S., Alexander S., Puccio A.M., Beers S.R., Ren D. (2014). Mitochondrial polymorphisms impact outcomes after severe traumatic brain injury. J. Neurotrauma.

[23] Fesharaki-Zadeh A., Datta D. (2024). An overview of preclinical models of traumatic brain injury (TBI): Relevance to pathophysiological mechanisms. Front. Cell Neurosci..

[24] Wu Q., Xia S.X., Li Q.Q., Gao Y., Shen X., Ma L., Zhang M.Y., Wang T., Li Y.S., Wang Z.F. (2016). Mitochondrial division inhibitor 1 (Mdivi-1) offers neuroprotection through diminishing cell death and improving functional outcome in a mouse model of traumatic brain injury. Brain Res..

[25] Wiley C.A., Bissel S.J., Lesniak A., Dixon C.E., Franks J., Beer Stolz D., Sun M., Wang G., Switzer R., Kochanek P.M. (2016). Ultrastructure of Diaschisis Lesions after traumatic brain injury. J. Neurotrauma.

[26] Hackett E.P., Chen J., Ingle L., Al Nemri S., Barshikar S., da Cunha Pinho M., Plautz E.J., Bartnik-Olson B.L., Park J.M. (2023). Longitudinal assessment of mitochondrial dysfunction in acute traumatic brain injury using hyperpolarized [1-^13^C]pyruvate. Magn. Reson Med..

[27] Xiong Y., Gu Q., Peterson P.L., Muizelaar J.P., Lee C.P. (1997). Mitochondrial dysfunction and calcium perturbation induced by traumatic brain injury. J. Neurotrauma.

[28] Gilmer L.K., Roberts K.N., Joy K., Sullivan P.G., Scheff S.W. (2009). Early mitochondrial dysfunction after cortical contusion injury. J. Neurotrauma.

[29] Harris J.J., Jolivet R., Attwell D. (2012). Synaptic energy use and supply. Neuron.

[30] Vos M., Lauwers E., Verstreken P. (2010). Synaptic mitochondria in synaptic transmission and organization of vesicle pools in health and disease. Front. Synaptic Neurosci..

[31] Hill R.L., Kulbe J.R., Singh I.N., Wang J.A., Hall E.D. (2018). Synaptic mitochondria are more susceptible to traumatic brain injury-induced oxidative damage and respiratory dysfunction than non-synaptic mitochondria. Neuroscience.

[32] Opii W.O., Nukala V.N., Sultana R., Pandya J.D., Day K.M., Merchant M.L., Klein J.B., Sullivan P.G., Butterfield D.A. (2007). Proteomic identification of oxidized mitochondrial proteins following experimental traumatic brain injury. J. Neurotrauma.

[33] Chen H., Chan Y.L., Nguyen L.T., Mao Y., de Rosa A., Beh I.T., Chee C., Oliver B., Herok G., Saad S. (2016). Moderate traumatic brain injury is linked to acute behaviour deficits and long term mitochondrial alterations. Clin. Exp. Pharmacol. Physiol..

[34] Dai W., Cheng H.L., Huang R.Q., Zhuang Z., Shi J.X. (2009). Quantitative detection of the expression of mitochondrial cytochrome c oxidase subunits mRNA in the cerebral cortex after experimental traumatic brain injury. Brain Res..

[35] Lin Y., Zhang J., Lu D., Zhang Y., Xu J., Wang S., Cheng X., Qin J., Zhang L., Li H. (2023). Uqcr11 alleviates oxidative stress and apoptosis after traumatic brain injury. Exp. Neurol..

[36] Yonutas H.M., Vekaria H.J., Sullivan P.G. (2016). Mitochondrial specific therapeutic targets following brain injury. Brain Res..

[37] Sullivan P.G., Rabchevsky A.G., Waldmeier P.C., Springer J.E. (2005). Mitochondrial permeability transition in CNS trauma: Cause or effect of neuronal cell death?. J. Neurosci. Res..

[38] Hubbard W.B., Vekaria H.J., Velmurugan G.V., Kalimon O.J., Prajapati P., Brown E., Geisler J.G., Sullivan P.G. (2023). Mitochondrial dysfunction after repeated mild blast traumatic brain injury Is attenuated by a mild mitochondrial uncoupling prodrug. J. Neurotrauma.

[39] Mantle D., Dewsbury M., Hargreaves I.P. (2024). The Ubiquinone-Ubiquinol redox cycle and Its clinical consequences: An overview. Int. J. Mol. Sci..

[40] Lee K.H., Cha M., Lee B.H. (2020). Neuroprotective effect of antioxidants in the brain. Int. J. Mol. Sci..

[41] Awasthi D., Church D.F., Torbati D., Carey M.E., Pryor W.A. (1997). Oxidative stress following traumatic brain injury in rats. Surg. Neurol..

[42] Tyurin V.A., Tyurina Y.Y., Borisenko G.G., Sokolova T.V., Ritov V.B., Quinn P.J., Rose M., Kochanek P., Graham S.H., Kagan V.E. (2000). Oxidative stress following traumatic brain injury in rats: Quantitation of biomarkers and detection of free radical intermediates. J. Neurochem..

[43] Yen H.C., Chen T.W., Yang T.C., Wei H.J., Hsu J.C., Lin C.L. (2015). Levels of F2-isoprostanes, F4-neuroprostanes, and total nitrate/nitrite in plasma and cerebrospinal fluid of patients with traumatic brain injury. Free Radic. Res..

[44] Gutierrez-Mariscal F.M., Arenas-de Larriva A.P., Limia-Perez L., Romero-Cabrera J.L., Yubero-Serrano E.M., López-Miranda J. (2020). Coenzyme Q10 supplementation for the reduction of oxidative tress: Clinical implications in the treatment of chronic diseases. Int. J. Mol. Sci..

[45] Corps K.N., Roth T.L., McGavern D.B. (2015). Inflammation and neuroprotection in traumatic brain injury. JAMA Neurol..

[46] Simon D.W., McGeachy M.J., Bayır H., Clark R.S., Loane D.J., Kochanek P.M. (2017). The far-reaching scope of neuroinflammation after traumatic brain injury. Nat. Rev. Neurol..

[47] Mantle D., Heaton R.A., Hargreaves I.P. (2021). Coenzyme Q10 and immune function: An overview. Antioxidants.

[48] Wong J., Hoe N.W., Zhiwei F., Ng I. (2005). Apoptosis and traumatic brain injury. Neurocrit. Care.

[49] Unnisa A., Greig N.H., Kamal M.A. (2023). Inhibition of Caspase 3 and Caspase 9 mediated apoptosis: A multimodal therapeutic target in traumatic brain injury. Curr. Neuropharmacol..

[50] Li X., Zhan J., Hou Y., Chen S., Hou Y., Xiao Z., Luo D., Lin D. (2019). Coenzyme Q10 suppresses oxidative stress and apoptosis via activating the Nrf-2/NQO-1 and NF-κB signaling pathway after spinal cord injury in rats. Am. J. Transl. Res..

[51] Sumi K., Okura T., Fujioka Y., Kato M., Imamura T., Taniguchi S.I., Yamamoto K. (2018). Coenzyme Q10 suppresses apoptosis of mouse pancreatic β-cell line MIN6. Diabetol. Metab. Syndr..

[52] Geng Z., Guo Z., Guo R., Ye R., Zhu W., Yan B. (2021). Ferroptosis and traumatic brain injury. Brain Res. Bull..

[53] Fikry H., Saleh L.A., Mahmoud F.A., Gawad S.A., Abd-Alkhalek H.A. (2024). CoQ10 targeted hippocampal ferroptosis in a status epilepticus rat model. Cell Tissue Res..

[54] Peng Z., Ding Y.N., Yang Z.M., Li X.J., Zhuang Z., Lu Y., Tang Q.S., Hang C.H., Li W. (2024). Neuron-targeted liposomal coenzyme Q10 attenuates neuronal ferroptosis after subarachnoid hemorrhage by activating the ferroptosis suppressor protein 1/coenzyme Q10 system. Acta Biomater..

[55] Li D., Zhang G., Wang Z., Guo J., Liu Y., Lu Y., Qin Z., Xu Y., Cao C., Wang B. (2023). Idebenone attenuates ferroptosis by inhibiting excessive autophagy via the ROS-AMPK-mTOR pathway to preserve cardiac function after myocardial infarction. Eur. J. Pharmacol..

[56] Avcı B., Günaydın C., Güvenç T., Yavuz C.K., Kuruca N., Bilge S.S. (2021). Idebenone ameliorates rotenone-induced parkinson’s disease in rats through decreasing lipid peroxidation. Neurochem. Res..

[57] Tao L., Xue Y.F., Sun F.F., He X., Wang H.Q., Tong C.C., Zhang C., Xu D.X., Chen X. (2024). MitoQ protects against carbon tetrachloride-induced hepatocyte ferroptosis and acute liver injury by suppressing mtROS-mediated ACSL4 upregulation. Toxicol. Appl. Pharmacol..

[58] Lazzarino G., Mangione R., Saab M.W., Tavazzi B., Pittalà A., Signoretti S., Di Pietro V., Lazzarino G., Amorini A.M. (2023). Traumatic brain injury alters cerebral concentrations and redox states of coenzymes Q9 and Q10 in the rat. Antioxidants.

[59] Kalayci M., Unal M.M., Gul S., Acikgoz S., Kandemir N., Hanci V., Edebali N., Acikgoz B. (2011). Effect of coenzyme Q10 on ischemia and neuronal damage in an experimental traumatic brain-injury model in rats. BMC Neurosci..

[60] Pierce J.D., Gupte R., Thimmesch A., Shen Q., Hiebert J.B., Brooks W.M., Clancy R.L., Diaz F.J., Harris J.L. (2018). Ubiquinol treatment for TBI in male rats: Effects on mitochondrial integrity, injury severity, and neurometabolism. J. Neurosci. Res..

[61] Pierce J.D., Shen Q., Peltzer J., Thimmesch A., Hiebert J.B. (2017). A pilot study exploring the effects of ubiquinol on brain genomics after traumatic brain injury. Nurs. Outlook.

[62] Salama A., Elgohary R. (2021). L-carnitine and Co Q10 ameliorate potassium dichromate -induced acute brain injury in rats targeting AMPK/AKT/NF-κβ. Int. Immunopharmacol..

[63] El-Laithy N.A., Mahdy E.M.E., Youness E.R., Shafee N., Mowafy M.S.S., Mabrouk M.M. (2018). Effect of Co Enzyme Q10 alone or in combination with Vitamin C on lipopolysaccharide-induced brain injury in rats. Biomed. Pharmacol. J..

[64] Haidar M.A., Shakkour Z., Barsa C., Tabet M., Mekhjian S., Darwish H., Goli M., Shear D., Pandya J.D., Mechref Y. (2022). Mitoquinone helps combat the neurological, cognitive, and molecular consequences of open head traumatic brain injury at chronic time point. Biomedicines.

[65] Tabet M., El-Kurdi M., Haidar M.A., Nasrallah L., Reslan M.A., Shear D., Pandya J.D., El-Yazbi A.F., Sabra M., Mondello S. (2022). Mitoquinone supplementation alleviates oxidative stress and pathologic outcomes following repetitive mild traumatic brain injury at a chronic time point. Exp. Neurol..

[66] Gülşen İ., Ak H., Çölçimen N., Alp H.H., Akyol M.E., Demir İ., Atalay T., Balahroğlu R., Rağbetli M.Ç. (2016). Neuroprotective effects of thymoquinone on the hippocampus in a rat model of traumatic brain injury. World Neurosurg..

[67] Suárez-Rivero J.M., Pastor-Maldonado C.J., Povea-Cabello S., Álvarez-Córdoba M., Villalón-García I., Munuera-Cabeza M., Suárez-Carrillo A., Talaverón-Rey M., Sánchez-Alcázar J.A. (2021). Coenzyme Q10 analogues: Benefits and challenges for therapeutics. Antioxidants.

[68] Wainwright L., Hargreaves I.P., Georgian A.R., Turner C., Dalton R.N., Abbott N.J., Heales S.J.R., Preston J.E. (2020). CoQ10 Deficient endothelial cell culture model for the investigation of CoQ10 blood-brain barrier transport. J. Clin. Med..

[69] Wu D., Chen Q., Chen X., Han F., Chen Z., Wang Y. (2023). The blood-brain barrier: Structure, regulation, and drug delivery. Signal Transduct. Target. Ther..

[70] Mantle D., Lopez-Lluch G., Hargreaves I.P. (2023). Coenzyme Q10 metabolism: A review of unresolved issues. Int. J. Mol. Sci..

[71] Mantle D., Hargreaves I.P. (2022). Mitochondrial dysfunction and neurodegenerative disorders: Role of nutritional supplementation. Int. J. Mol. Sci..

[72] Mantle D., Heaton R.A., Hargreaves I.P. (2021). Coenzyme Q10, ageing and the nervous system: An overview. Antioxidants.

[73] Pardridge W.M. (2012). Drug transport across the blood-brain barrier. J. Cereb. Blood Flow. Metab..

[74] Dhuria S.V., Hanson L.R., Frey W.H. (2010). Intranasal delivery to the central nervous system: Mechanisms and experimental considerations. J. Pharm. Sci..

[75] Crowe T.P., Greenlee M.H.W., Kanthasamy A.G., Hsu W.H. (2018). Mechanism of intranasal drug delivery directly to the brain. Life Sci..

[76] Pandya J.D., Musyaju S., Modi H.R., Okada-Rising S.L., Bailey Z.S., Scultetus A.H., Shear D.A. (2024). Intranasal delivery of mitochondria targeted neuroprotective compounds for traumatic brain injury: Screening based on pharmacological and physiological properties. J. Transl. Med..

[77] Tepper S.J., Cady R.K., Silberstein S., Messina J., Mahmoud R.A., Djupesland P.G., Shin P., Siffert J. (2015). AVP-825 breath-powered intranasal delivery system containing 22 mg sumatriptan powder vs. 100 mg oral sumatriptan in the acute treatment of migraines (The COMPASS study): A comparative randomized clinical trial across multiple attacks. Headache.

[78] Lipton R.B., Munjal S., Buse D.C., Bennett A., Fanning K.M., Burstein R., Reed M.L. (2017). Allodynia Is Associated With Initial and Sustained Response to Acute Migraine Treatment: Results from the American Migraine Prevalence and Prevention Study. Headache.

[79] Lipton R.B., Munjal S., Alam A., Buse D.C., Fanning K.M., Reed M.L., Schwedt T.J., Dodick D.W. (2018). Migraine in America Symptoms and Treatment (MAST) Study: Baseline Study Methods, Treatment Patterns, and Gender Differences. Headache.

[80] Claxton A., Baker L.D., Hanson A., Trittschuh E.H., Cholerton B., Morgan A., Callaghan M., Arbuckle M., Behl C., Craft S. (2015). Long-acting intranasal insulin detemir improves cognition for adults with mild cognitive impairment or early-stage Alzheimer’s disease dementia. J. Alzheimers Dis..

[81] Kellar D., Lockhart S.N., Aisen P., Raman R., Rissman R.A., Brewer J., Craft S. (2021). Intranasal Insulin Reduces White Matter Hyperintensity Progression in Association with Improvements in Cognition and CSF Biomarker Profiles in Mild Cognitive Impairment and Alzheimer’s Disease. J. Prev. Alzheimers Dis..

[82] Kellar D., Register T., Lockhart S.N., Aisen P., Raman R., Rissman R.A., Brewer J., Craft S. (2022). Intranasal insulin modulates cerebrospinal fluid markers of neuroinflammation in mild cognitive impairment and Alzheimer’s disease: A randomized trial. Sci. Rep..

[83] Bancke L.L., Dworak H.A., Rodvold K.A., Halvorsen M.B., Gidal B.E. (2015). Pharmacokinetics, pharmacodynamics, and safety of USL261, a midazolam formulation optimized for intranasal delivery, in a randomized study with healthy volunteers. Epilepsia.

[84] Milési C., Baleine J., Mura T., Benito-Castro F., Ferragu F., Thiriez G., Thévenot P., Combes C., Carbajal R., Cambonie G. (2018). Nasal midazolam vs ketamine for neonatal intubation in the delivery room: A randomised trial. Arch. Dis. Child. Fetal Neonatal Ed..

[85] Henney III H.R., Sperling M.R., Rabinowicz A.L., Bream G., Carrazana E.J. (2014). Assessment of pharmacokinetics and tolerability of intranasal diazepam relative to rectal gel in healthy adults. Epilepsy Res..

[86] Ahmad S., Ellis J.C., Kamwendo H., Molyneux E. (2006). Efficacy and safety of intranasal lorazepam versus intramuscular paraldehyde for protracted convulsions in children: An open randomised trial. Lancet.

[87] McIntyre R.S., Bitter I., Buyze J., Fagiolini A., Godinov Y., Gorwood P., Ito T., Oliveira-Maia A.J., Vieta E., Werner-Kiechle T. (2024). Safety and tolerability of esketamine nasal spray versus quetiapine extended release in patients with treatment resistant depression. Eur. Neuropsychopharmacol..

[88] Vitale D.C., Piazza C., Sinagra T., Urso V., Cardì F., Drago F., Salomone S. (2013). Pharmacokinetic characterization of tizanidine nasal spray, a novel intranasal delivery method for the treatment of skeletal muscle spasm. Clin. Drug Investig..

[89] Simmons R.G., Phillips J.B., Lojewski R.A., Wang Z., Boyd J.L., Putcha L. (2010). The efficacy of low-dose intranasal scopolamine for motion sickness. Aviat Space Environ. Med..

[90] Silachev D.N., Plotnikov E.Y., Zorova L.D., Pevzner I.B., Sumbatyan N.V., Korshunova G.A., Gulyaev M.V., Pirogov Y.A., Skulachev V.P., Zorov D. (2015). Neuroprotective effects of mitochondria-targeted plastoquinone and thymoquinone in a rat model of brain ischemia/reperfusion injury. Molecules.

[91] Kauli R., Laron Z. (1974). A vasopressin analogue in treatment of diabetes insipidus. Arch. Dis. Child..

[92] Kalra S., Dhingra M. (2019). Intranasal glucagon. J. Pak. Med. Assoc..

[93] Priya G., Kalra S., Dasgupta A., Grewal E. (2021). Diabetes Insipidus: A pragmatic approach to management. Cureus.

[94] Hasanloei M.A.V., Zeinaly A., Rahimlou M., Houshyar H., Moonesirad S., Hashemi R. (2021). Effect of coenzyme Q10 supplementation on oxidative stress and clinical outcomes in patients with low levels of coenzyme Q10 admitted to the intensive care unit. J. Nutr. Sci..

[95] Hargreaves I.P., Mantle D. (2019). Supplementation with selenium and coenzyme Q10 in critically ill patients. Br. J. Hosp. Med..

[96] Mantle D., Dybring A. (2020). Bioavailability of Coenzyme Q10: An overview of the absorption process and subsequent metabolism. Antioxidants.

[97] Kalenikova E.I., Gorodetskaya E.A., Povarova O.V., Medvedev O.S. (2024). Prospects of Intravenous Coenzyme Q10 Administration in Emergency Ischemic Conditions. Life.

[98] Cash A., Theus M.H. (2020). Mechanisms of blood-brain barrier dysfunction in traumatic brain injury. Int. J. Mol. Sci..

[99] Capossela L., Graglia B., Ferretti S., Di Sarno L., Gatto A., Calcagni M.L., Di Giuda D., Cocciolillo F., Romeo D.M., Manni L. (2024). Intranasal human-recombinant nerve growth factor administration improves cognitive functions in a child with severe traumatic brain injury. Eur. Rev. Med. Pharmacol. Sci..

[100] Huang C.H., Yang C.T., Chang C.C. (2023). Traumatic brain injury and risk of heart failure and coronary heart disease: A nationwide population-based cohort study. PLoS ONE.

[101] Zhao Q., Yan T., Li L., Chopp M., Venkat P., Qian Y., Li R., Wu R., Li W., Lu M. (2019). Immune response mediates cardiac dysfunction after traumatic brain injury. J. Neurotrauma.

[102] Mantle D. (2015). Coenzyme Q10 and cardiovascular disease: An overview. Br. J. Cardiol..

[103] Zhu X., Cheng J., Yu J., Liu R., Ma H., Zhao Y. (2023). Nicotinamide mononucleotides alleviated neurological impairment via anti-neuroinflammation in traumatic brain injury. Int. J. Med. Sci..

[104] Toklu H.Z., Hakan T., Biber N., Solakoğlu S., Oğünç A.V., Sener G. (2009). The protective effect of alpha lipoic acid against traumatic brain injury in rats. Free Radic. Res..

[105] Hiskens M.I., Li K.M., Schneiders A.G., Fenning A.S. (2023). Repetitive mild traumatic brain injury-induced neurodegeneration and inflammation is attenuated by acetyl-L-carnitine in a preclinical model. Front. Pharmacol..

